# Comparative study of the most commonly used methods for total protein determination in milk of different species and their ultrafiltration products

**DOI:** 10.3389/fnut.2022.925565

**Published:** 2022-09-13

**Authors:** Diego Hueso, Javier Fontecha, Pilar Gómez-Cortés

**Affiliations:** Department of Bioactivity and Food Analysis, Institute of Food Science Research (CIAL, CSIC-UAM), Madrid, Spain

**Keywords:** ultrafiltered milk, whey protein, Kjeldahl, Dumas, BCA, Bradford, SDS-PAGE

## Abstract

Milk ultrafiltration is a widely used membrane filtration process that allows the recuperation of whey proteins in a concentrate high in total solids, which can later be transformed in multiple healthy dairy products with great prospects for the food industry. Protein content is a decisive factor for the technological performance of milk concentrates and currently, the ISO standard method for its determination is Kjeldahl, which is time-consuming and requires specific instrumentation. For this reason, the use of rapid methods to quantify protein would greatly facilitate the monitoring of the milk ultrafiltration process. In this study, the bicinchoninic acid assay (BCA), the detergent compatible Bradford assay and the Dumas method were compared to Kjeldahl protein determination to select a quick and accurate methodology suitable for milk of different species and its ultrafiltration products (retentates and permeates). The protein content obtained from Bradford assay and Dumas method in origin milk and retentate samples was consistent with Kjeldahl values. In contrast, BCA protein levels were significantly different when compared to Kjeldahl and no method was proved to be suitable for protein determination in permeate samples. The use of sodium dodecyl sulfate was also examined to improve protein measurements without success. In comparison with the official method, Bradford assay quantitatively provided the best results, and it would be recommended for a quick, economic and easy determination of total protein content in milk and retentate samples.

## Introduction

Milk is defined as the normal mammal secretion obtained by milking one or more times, without any type of addition or extraction of substances (1). It is a complex food containing a good balance between major nutrients (proteins, fat, and carbohydrates) and also rich in minerals and vitamins. Milk furnishes a broad range of nutritionally relevant compounds such as caseins, whey proteins, bioactive fatty acids, polar lipids and other minor constituents, which have functional properties both physiologically and technologically (2). Depending on the animal species, milk shows differences on its composition, from milk fat globule size to protein concentration. Each composition factor contributes to milk technological performance and the final characteristics of dairy products (3).

Whey proteins have aroused great interest not only for their physiological properties, but specially for being a by-product of the cheese industry produced in massive quantities (4). There are multiple approaches for the valorization and recovery of whey components, being membrane processes the most widely used in the dairy industry (5). The ultrafiltration (UF) systems concentrates caseins, whey proteins, total solids and colloidal salts in proportion to the amount of permeate removed (6, 7). Then, ultrafiltered milk can be processed and transformed into a wide variety of high-protein dairy products. The ISO standard method for quantifying the protein content of milk and milk products is Kjeldahl digestion, which consists of the determination of total nitrogen by oxidation of the sample with sulfuric acid and subsequent titration of ammonium sulphate with NaOH (8). However, this method is complex, time-consuming, and requires specific instrumentation and contaminant agents. Moreover, Kjeldahl typically uses large sample volumes and it is a destructive procedure. The Dumas method also determines the total nitrogen of the sample by combustion, but this methodology is faster and simpler than Kjeldahl, not requiring toxic chemicals. Dumas and Kjeldahl determinations may give rise to different results depending on the non-protein nitrogen content of the analyzed sample, since these methods are not capable of distinguishing non-protein nitrogen from protein nitrogen (9). Therefore, both methods are susceptible to interferences by organic and inorganic compounds containing nitrogen (9, 10).

In recent years, the alternative of using colorimetric assays for protein determination has spread widely because they are fast, easy to use and require a small amount of sample. There are two main colorimetric methods that differ in their basis: the detergent compatible Bradford assay that relies on the binding of the dye Coomassie Blue G250 to protein (11) and the bicinchoninic acid (BCA) assay, based on the Biuret reaction and cupper ion reduction in alkaline conditions (12). Although BCA is extensively used in food analysis to determine total protein content, non-accurate results have been observed in complex matrices (13, 14). In this line, it has been reported that reducing sugars (15) and phospholipids (16) can interfere with the Biuret reaction. Regarding dairy samples, it has also been observed that thermal treatments may affect the BCA protein measurements, which has been related to reducing substances originated during the heating processes of milk (17). These reducing substances would also be present in milk UF products due to the heat-treatment that occurs in both milk pasteurization and dairy products manufacture. On the other hand, the composition of the food matrix could also affect protein determination in the Bradford assay. Gazzola et al. (18) observed that the protein content in wine samples was underestimated due to the presence of ethanol and polyphenols. Other substances commonly used in electrophoresis, such as sodium dodecyl sulfate (SDS) or Triton-X100, have also been reported to interfere with the Bradford assay (19). In contrast, several modifications to the Bradford assay have been attempted to achieve correct protein determinations for specific samples like collagen, gels or plant proteins (20, 21).

The analytical methodology used to determine the protein content in milk and final dairy products must be precise and accurate, since total protein concentration can modify the technological performance of milk (22). In the present research, a comparative study between 4 different total protein determination methods (i.e., Kjeldahl, Dumas, BCA and Bradford) was carried out in milk from cows, goats and sheep and their UF products. Samples covered a wide range of protein concentrations and results were compared to the official method in order to select an accurate, quick and quantitative method that could be used for protein monitoring in the dairy industry. In addition, to our knowledge, this is the first comparative study that evaluates the addition of SDS as a mean to troubleshoot inaccuracy in protein determination by rapid colorimetric methods in dairy matrices. Milk, retentates and permeates were also analyzed by SDS polyacrylamide gel electrophoresis (SDS-PAGE) to control the UF process and determine which proteins were retained or lost within the permeate.

## Materials and methods

### Materials

Whole pasteurized milk from cow, goat and sheep were purchased in local supermarkets (Madrid, ES). Pierce^TM^ BCA Protein Assay Kit and Pierce^TM^ Detergent Compatible Bradford Assay Kit were purchased from ThermoFisher Scientific (Rockford, IL). SDS and BluSafe dye were acquired from Sigma-Aldrich (St. Louis, MO) and Nzytech (Lisbon, PT), respectively. Criterion^TM^ XT precast gels with 12% Bis-Tris, sample buffer, Precision Prestained Protein Dual Xtra Standard (250 kDa to 2kDa) and Bis-Tris SDS running buffer were obtained from Bio-Rad Laboratories (Hercules, CA).

### Skimming of milk and ultrafiltration

Milk skimming was carried out by centrifugation at 9000 rpm for 30 min at 25°C. Whole and skimmed milks were ultrafiltered using a Centramate^TM^ 500 S Tangencial Flow Filtration System. The UF was performed in duplicate using a minimum protein binding polyethersulphone membrane with a pore size of 30 kDa and a mean transmembrane pressure (TMP) of 0.25 bar. To obtain similar concentration factors, the UF process was always carried out with 1 L of milk and it was stopped when 570 mL of permeate were measured in a graduated cylinder. Original milks and their UF products (retentates and permeates) were collected in falcon tubes and kept at −20°C until analysis. Before protein determination, samples were thawed in a thermoblock to 40°C and then well mixed using a vortex.

### Electrophoresis

SDS-PAGE assay was performed as described in Villas-Boas et al. (23). Briefly, samples were diluted with PBS to achieve a protein concentration of 4 mg/mL. Then, sample dilutions were well-mixed with sample buffer to a 1:4 ratio (sample:buffer). After heating samples at 95°C for 5 min, samples were analyzed on a precast Criterion XT 12% Bis-Tris gel, through a separation carried out at 120 V. Bands were finally stained directly with BlueSafe dye for 1 h.

### Colorimetric assays for protein determination

The BCA assay was performed in 96-well microplates following the user guide. Briefly, 25 μl of each sample and 200 μl of BCA working reagent were added to each well. The microplate was shaken for 15 seconds and then incubated for 30 min at 37°C using a BioTek^®^ Cytation 5 Cell Imaging Multi-Mode Reader. Absorbance was measured at 562 nm and protein concentration in samples was determined by interpolation on the bovine serum albumin (BSA) standard curve.

The Bradford assay was performed in 96-well microplates following the user guide. Briefly, 10 μl of each sample and 300 μl of Coomassie reagent were added to each well. After 10 min incubation at room temperature, absorbance was measured at 595 nm using the same BioTek^®^ Cytation 5 Cell Imaging Multi-Mode Reader and protein concentration in samples was determined by interpolation on the BSA standard curve.

Both colorimetric assays were performed in triplicate on three different days (*n* = 9). Sample dilutions and standard curves were made with MilliQ water. When SDS was used, both samples and standard curves were diluted with 2% SDS in MilliQ water since both Bradford and BCA kits are detergent resistant. Table 1 summarizes the main advantages and disadvantages of using colorimetric methods for protein determination.

**Table 1:** Advantages and disadvantages of the most commonly used methods for total protein determination.

**Method**	**LOQ^a^ (μg/mL)**	**Basis**	**Advantages**	**Disadvantages**
Kjeldahl	500	Total nitrogen determination by sulfuric acid digestion and ammonium titration	· Robust technique suitable for different sample matrices · Official ISO method for protein quantification in milk and dairy products	· Chemicals needed · Specific instrumentation required · Long analysis time · Indirect measurement of total protein that requires a matrix-dependent correction factor
Dumas	100	Total nitrogen determination by high temperature combustion and inorganic nitrogen detection	· No chemicals needed · High correlation to Kjeldahl determinations · Fast analysis · High level of automation	· Specific instrumentation required · Indirect measurement of total protein that requires a matrix-dependent correction factor
Bradford	100	Protein determination by Coomassie dye-binding and absorbance measurement	· Simple, easy, fast and cost-effective analysis · No specific equipment is required · Small amount of sample · Direct measurement of protein content ·Not affected by non-protein nitrogen	· Protein determination is influenced by the presence of common protein surfactants · Quantification sensitive to amino acid composition
BCA	20	Protein determination by biuret reaction and absorbance measurement	· Simple, easy, fast and cost-effective analysis · No specific equipment is required · Small amount of sample · Direct measurement of protein content · High sensitivity · Wide detection range	· Protein determination is influenced by reducing agents and chelators · Colorimetric reaction does not have an end-point

a LOQ, Limit of quantification.

### Dumas and Kjeldahl methods for protein determination

The Dumas method was performed in a LECO Corporation TruMac (St. Joseph, MI) in duplicate. One mL of sample was combusted at 1100°C under oxygen atmosphere and the gas was drawn by a He flux through filters and a cooler to remove water and particles. Then, a 10 mL aliquot was passed through a catalytic filter where NO_x_ are reduced to inorganic nitrogen and CO_2_ is removed by adsorption. Finally, the inorganic nitrogen reached the heat conducting cell and the electronic signal (measured as area) was compared to pure helium flux.

Kjeldahl method was carried out in duplicate, following the Standard ISO (8). Briefly, samples were homogenized with sulfuric acid and digested at 420°C in a digestion system that neutralizes fumes. After digestion, ammonium was distilled and titrated, providing the total nitrogen content of the sample. Total protein was calculated using the correction factor of milk (6.38). Table 1 summarizes the main advantages and disadvantages of using Dumas and Kjeldahl methods for protein determination.

### Statistical analysis

Statistical analysis was performed with GraphPad Prism software (San Diego, CA). A two-way Anova test was used to compare the protein content obtained by Kjeldahl with the other methods used. Multiple pairwise comparisons were carried out when SDS was used for troubleshooting tests (Section Use of SDS for troubleshooting). Differences were considered as statistically significant at *P* < 0.05.

## Results and discussion

### Ultrafiltration process and protein recovery

Table 2 shows the total protein content of milks and their corresponding retentates as determined by the official Kjeldahl method. During the UF process of milk, a theoretical volumetric concentration factor (VCF) is usually calculated, which indicates the expected concentration of total solids and other molecules larger than the pore size of the UF membrane. To evaluate the UF performance, an experimental protein concentration factor (PCF) was also calculated based on protein concentration (Table 2). Small milk components (e.g., lactose) are able to pass through the membrane pore and do not have a concentration value near the theoretical VCF. The pore size used in the present research (30 KDa) was selected to retain whey proteins in the retentate fraction. Thus, a correct performance of the UF process would be proven if calculated PCF is similar to the VCF value. All PCF (Table 2) were close to the theoretical VCF value (2.33). On the other hand, the protein content of whole and skimmed milks did not differ substantially. Comparing between species, sheep's milk and sheep's milk retentate showed the highest protein contents (Table 2).

**Table 2:** Total protein content (g/100 mL) of cow, goat and sheep milk, before and after ultrafiltration (UF), determined by Kjeldahl.

			**Average ±SD^a^**	**PCF^b^**
COW	Skimmed	Starting milk	3.26 ± 0.03	2.38
		Retentate	7.75 ± 0.66	
	Whole	Starting milk	3.36 ± 0.02	2.36
		Retentate	7.92 ± 0.03	
GOAT	Skimmed	Starting milk	3.04 ± 0.03	2.44
		Retentate	7.41 ± 0.03	
	Whole	Starting milk	3.56 ± 0.01	2.25
		Retentate	8.01 ± 0.16	
SHEEP	Skimmed	Starting milk	5.27 ± 0.02	1.84
		Retentate	9.68 ± 0.06	
	Whole	Starting milk	5.28 ± 0.08	2.10
		Retentate	11.09 ± 0.03	

a Each value is obtained from two UF performed on different days and duplicate Kjeldahl analysis.

b PCF, Protein Concentration Factor.

**Figure 1:**
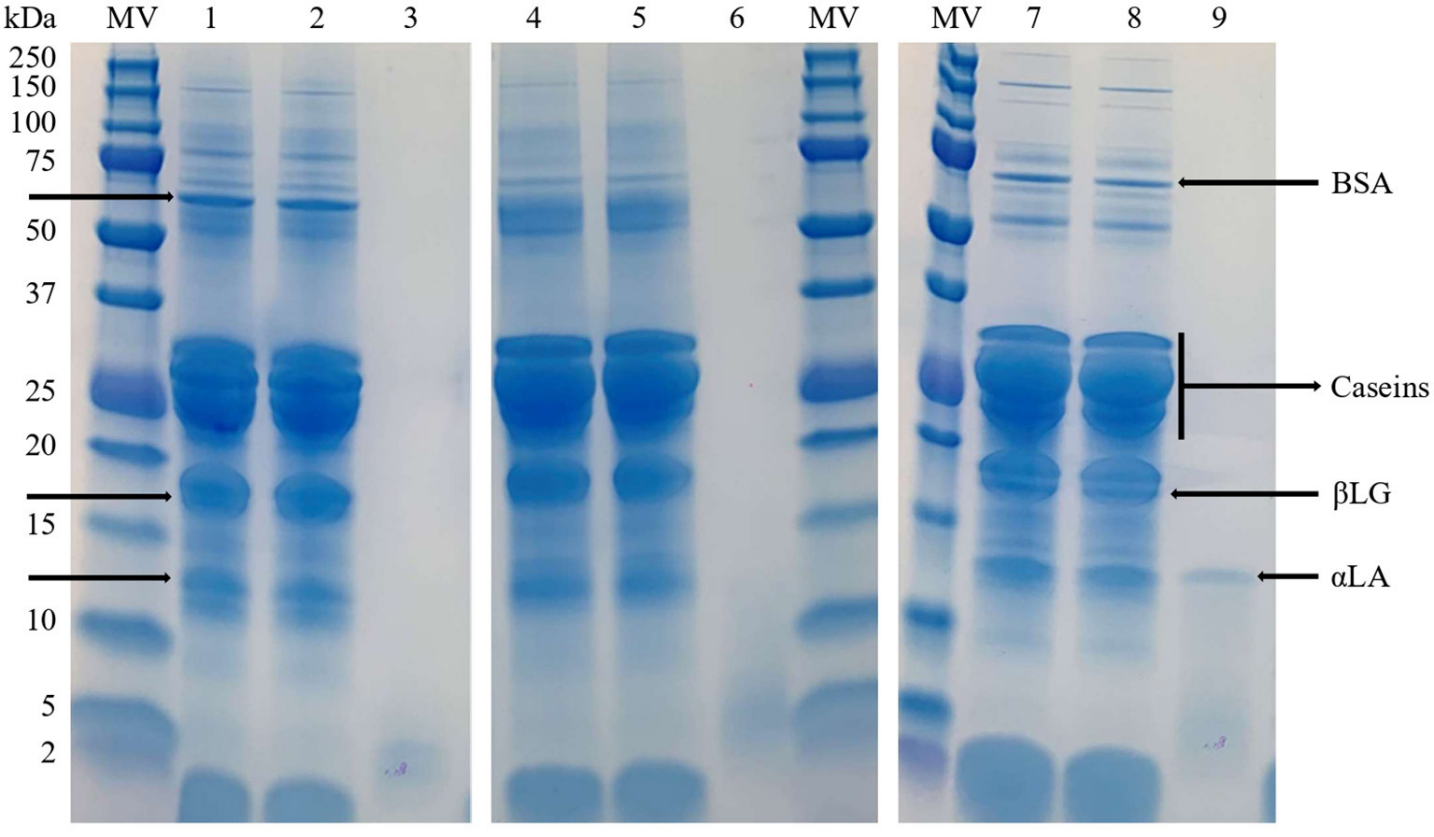
SDS-PAGE analysis of cow milk (1), cow milk retentate (2), cow milk permeate (3), goat milk (4), goat milk retentate (5), goat milk permeate (6), sheep milk (7), sheep milk retentate (8) and sheep milk permeate (9). MW, Molecular weight standard; αLA, α-lactalbumin; βLG, β-lactoglobulin; BSA, bovine serum albumin.

The SDS-PAGE assay confirmed that whey proteins were successfully retained in milk retentates by UF with a 30 kDa membrane (Figure 1). The protein profiles of the retentates were identical to their respective original milks for all ruminant species. It is important to note that these UF retentates, which will be transformed into dairy products by coagulation, preserve high-value whey proteins such as bovine serum albumin, β-lactoglobulin and α-lactalbumin. Regarding milk permeates, their protein profiles differed greatly from cheese whey derived from traditional milk coagulation (24). Electrophoresis of sheep's milk permeates, from both whole and skimmed milks, revealed a band between 10–15 kDa that corresponds to α-lactalbumin. It would indicate that α-lactalbumin is able to pass through the membrane pores, but it was not detected in cow's or goat's permeates due to the higher relative concentration of α-lactalbumin in the original sheep's milk samples. To completely retain whey proteins, the membrane pore size must be at least smaller than the size of α-lactalbumin, considering the normal fluctuation of protein size. The bands below 5 kDa (Figure 1) would be related to peptides from protein degradation naturally occurring in milk (25, 26).

### Comparison of total protein determination methods in milk

**Figure 2:**
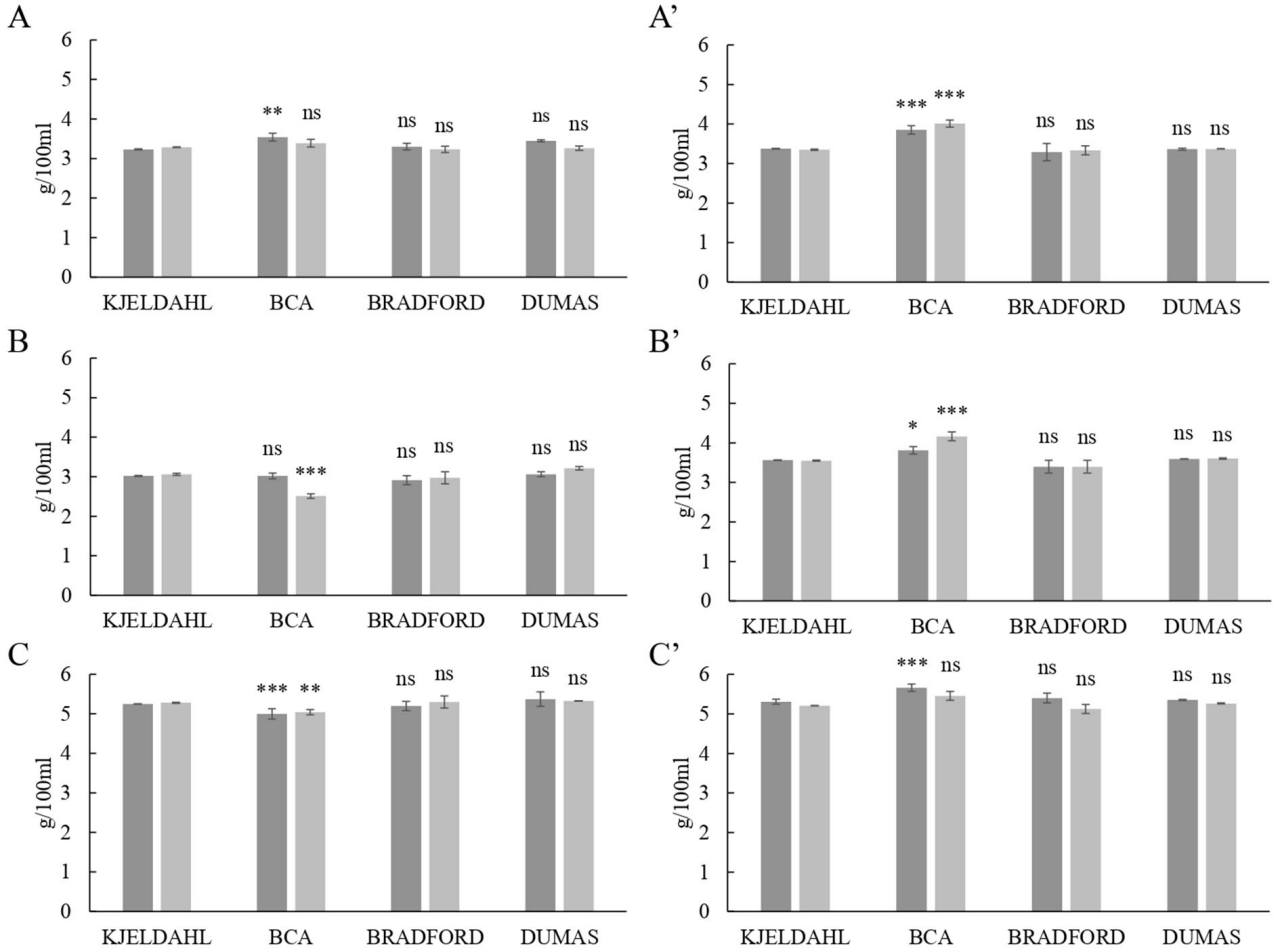
Comparison of protein levels in milk (g / 100 mL) obtained by Kjeldahl official reference method with the contents determined by the bicinchoninic acid assay (BCA), the detergent compatible Bradford assay and the Dumas method. **(A)** Cow's skimmed milk, **(A')** cow's whole milk, **(B)** goat's skimmed milk, **(B')** goat's whole milk, **(C)** sheep's skimmed milk, and **(C')** sheep's whole milk. Different shades of gray indicate different ultrafiltration processes. *P*-value: ^***^ = *P* < 0.001; ^**^ = *P* < 0.01; ^*^ = *P* < 0.05; ns = *P* > 0.05.

Total protein contents in skimmed and whole milks from the three ruminant species determined by Dumas and colorimetric assays were compared with the Kjeldahl method (Figure 2). Even though duplicates of UF were performed with the same type of milk, each liter was processed at different days so original milk samples from UF1 were not mixed with the ones from UF2 and they were analyzed separately. The Bradford and Dumas determinations were not significantly different from the Kjeldahl value, in agreement with previous research. For instance, Wiles et al. (27) reported that there is no evidence for a generic difference between Dumas and Kjeldal methods for multiple dairy products. In addition, Kamizake et al. (28) established that the Bradford assay could be used for the direct determination of total protein content in reconstituted whole and skimmed milks. Our results would also indicate that the Bradford colorimetric method successfully determined the total protein content in pasteurized milk samples with different levels of fat and total solids. In contrast, BCA protein determinations were significantly different from Kjeldahl (*P* < 0.05) in every pasteurized whole milk sample, regardless of the species (Figure 2).

BCA assays are widely used for the determination of total protein content in food and biological samples. However, there is some controversy regarding the reliability of BCA results. Keller and Neville (29) observed that the BCA assay was the most consistent and reliable method for determination of total protein in milk compared to Kjeldahl and, more recently, Giuffrida et al. (30) reported that the total protein content determined by BCA was not significantly different from the Kjeldahl value in human milks. In contrast, Lonnerdal et al. (31) showed that the BCA assay consistently overestimated Kjeldahl protein values by 30%. Bergqvist et al. (32) determined that lactose would contribute to an overestimation of up to 15% of the total protein content when using the BCA assay in human milk. The results presented in Figure 2 indicate that the BCA methodology is not reliable for the quantitative determination of total protein in milk samples.

### Comparison of total protein determination methods in milk UF products

**Figure 3:**
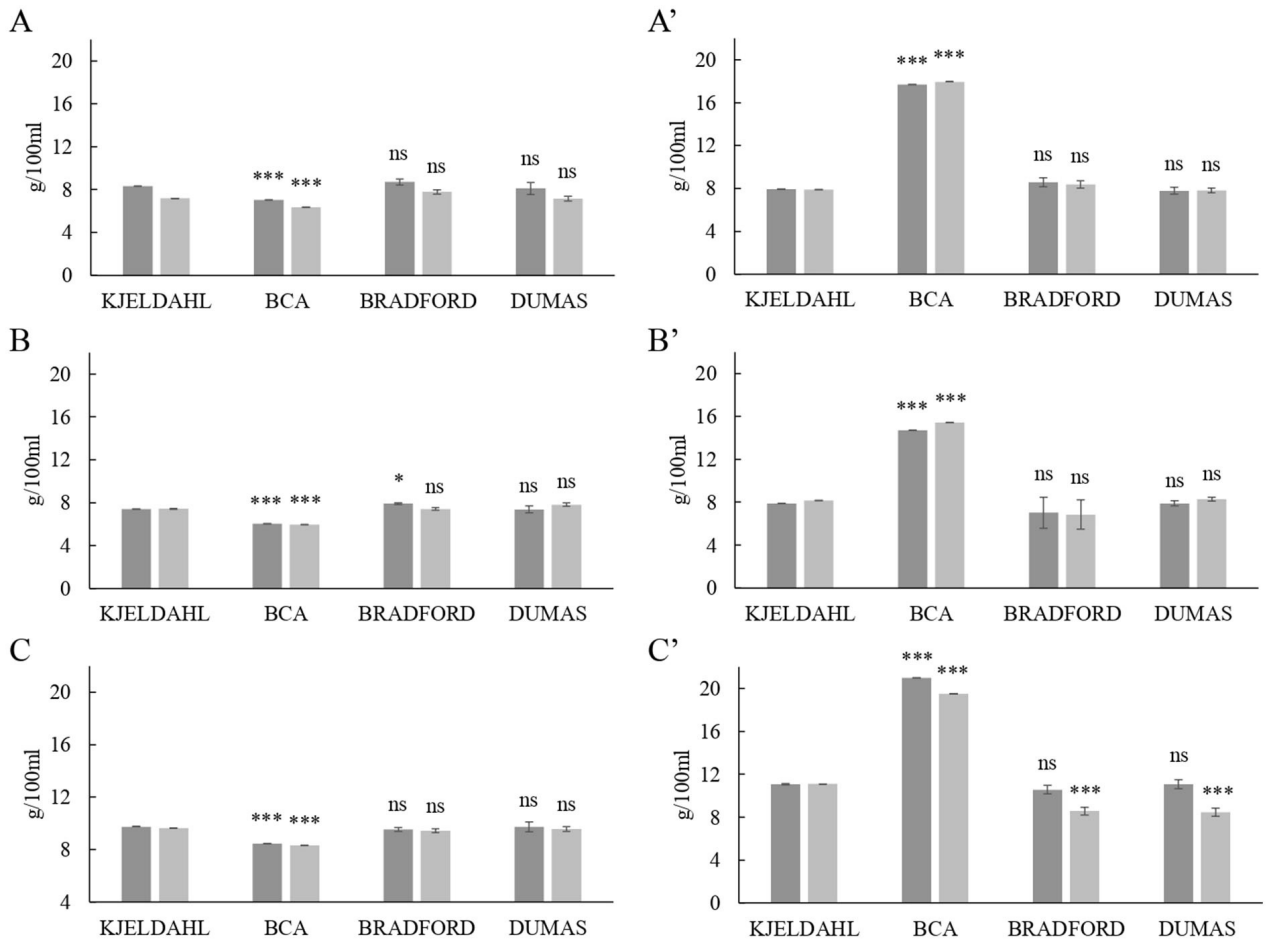
Comparison of protein levels in milk retentate (g / 100 mL) obtained by Kjeldahl official reference method with the contents determined by the bicinchoninic acid assay (BCA), the detergent compatible Bradford assay and the Dumas method. **(A)** Cow's skimmed retentate, **(A')** cow's whole retentate, **(B)** goat's skimmed retentate, **(B')** goat's whole retentate, **(C)** sheep's skimmed retentate, and **(C')** sheep's whole retentate. Different shades of gray indicate different ultrafiltration processes. *P*–value: *** = *P* < 0.001; ** = *P* < 0.01; * = *P* < 0.05; ns = *P* > 0.05.

The comparison of the total protein contents in milk retentates after UF is displayed in Figure 3. Similar to the results obtained in the original milk samples, Bradford and Dumas quantifications did not substantially differ from those of Kjeldahl, although a slight decrease was observed in sheep's whole retentate. However, when comparing BCA assay to Kjeldahl, the protein contents for skimmed and whole milk retentates were significantly different. The BCA underestimated protein content in skimmed milk retentates from all species, but it was overestimated on average by 2-fold in whole milk retentates samples (Figure 3). For instance, cow's whole retentate determined by BCA showed a total protein content of 17.81 ± 0.40 g/100 mL, which was significantly different to Kjeldahl determination 7.92 ± 0.03 g/100 mL. These results confirm that the dairy matrix affects BCA determination and that milk fat would interfere in total protein quantification. It is important to note that milk fat is dispersed in the form of triglyceride globules covered by a lipid trilayer membrane rich in phospholipids (33). Those phospholipids would interfere with the BCA Biuret reaction (16) and thus the assay would overestimate protein content. This would explain the high protein contents obtained in the retentates from whole milk, which are underestimated in skimmed milk retentates (Figure 3). These differences in protein determination, when compared to Kjeldahl, indicate that the BCA assay is not suitable for concentrated samples such as milk retentates.

For milk permeates, the protein values were very different depending on the method used. The protein contents obtained by the Kjeldahl reference method were in the range of 0.138–0.273 g per 100 mL of permeate (Table 3). Despite this, the Bradford assay failed to determine protein levels as the values obtained were below the detection limit (100 μg/mL, LOD). Dumas analysis was not suitable to determine the protein content in milk permeates, since the results obtained were overestimated and significantly different from Kjeldahl (Table 3). BCA protein concentrations were more similar to Kjeldahl than the other methods tested, which would be related to the absence of fat in milk permeates.

**Table 3:** Comparison of total protein content in permeate samples (g/100 mL) determined by Kjeldahl official reference method with the bicinchoninic acid assay (BCA), the detergent compatible Bradford assay and the Dumas method.

		**Kjeldahl**	**BCA**	**Bradford**	**Dumas**
COW	Skimmed	0.14 ± 0.02	0.15 ± 0.02	<LOD	0.21 ± 0.01^***^
	Whole	0.16 ± 0.04	0.15 ± 0.02	<LOD	0.21 ± 0.16
GOAT	Skimmed	0.23 ± 0.01	0.16 ± 0.02^***^	<LOD	0.27 ± 0.01^***^
	Whole	0.18 ± 0.02	0.16 ± 0.02	<LOD	0.25 ± 0.05^**^
SHEEP	Skimmed	0.25 ± 0.07	0.22 ± 0.02	<LOD	0.28 ± 0.07
	Whole	0.27 ± 0.02	0.24 ± 0.02^*^	<LOD	0.37 ± 0.02^***^

P–value:

*** = P < 0.001;

** = P < 0.01;

* = P < 0.05 (Indicates significant differences with Kjeldahl reference value).

< LOD, Values lower than the limit of detection.

Kjeldahl reference method does not directly determine protein and it uses a matrix-dependent correction factor to quantify protein. For this reason, and since there is no specific factor for milk permeates, the protein content determined by Kjeldahl could be overestimated. It has been shown that the Kjeldahl method is not capable of detecting melamine adulteration in milk (10), thus amino acids and other non-protein nitrogen naturally present in milk permeates could be incorrectly measured as protein nitrogen when applying the correction factor. It is well known that the Bradford assay would not interfere with non-protein nitrogen (17, 34). The inability to determine the protein levels in milk permeates by Bradford (i.e., values < LOD, Table 3), together with the absence of bands in the electrophoresis gel (Figure 1), indicates that protein contents in permeates were at trace levels and none of the methods tested provided reliable results for this UF product.

### Use of SDS for troubleshooting

The determination of total protein content in food samples using the BCA assay is a widespread methodology in food science and technology research (35–37). The inaccurate results obtained for milk and UF products were attempted to be solved with SDS, as previously described by Morton and Evans (38). These authors overcame BCA assay overestimations by the addition of 2% SDS and we followed a similar approach in both colorimetric assays (Table 4). Results were not satisfactory as the addition of 2 % SDS not only did not improve BCA protein quantification, but it also worsened Bradford determination when compared to Kjeldahl reference method (Table 4).

**Table 4:** Total protein content (g/100 mL) determined by colorimetric assays, with and without sodium dodecyl sulfate (SDS), and Kjeldahl in milk samples and ultrafiltration retentates from cow, goat and sheep milks.

		**Kjeldahl**		**BCA**		**BCA + 2% SDS**		**Bradford**		**Bradford + 2% SDS**	
		**Milk**	**Retentate**	**Milk**	**Retentate**	**Milk**	**Retentate**	**Milk**	**Retentate**	**Milk**	**Retentate**
COW	Skimmed	3.26 ± 0.03 ^a^	7.75 ± 0.66 ^A^	2.90 ± 0.10 ^b^	6.69 ± 0.42 ^B^	2.63 ± 0.11 ^c^	5.50 ± 0.39 ^C^	3.26 ± 0.09 ^a^	8.09 ± 0.53 ^A^	4.16 ± 0.12 ^d^	9.77 ± 0.42 ^D^
	Whole	3.36 ± 0.02 ^a^	7.92 ± 0.03 ^A^	3.10 ± 0.08 ^b^	17.81 ± 0.40 ^B^	2.74 ± 0.14 ^c^	6.32 ± 0.37 ^C^	3.23 ± 0.20 ^ab^	8.38 ± 0.29 ^A^	4.64 ± 0.23 ^d^	10.94 ± 0.14 ^D^
GOAT	Skimmed	3.04 ± 0.03 ^a^	7.41 ± 0.03 ^A^	2.71 ± 0.10 ^b^	6.00 ± 0.18 ^B^	2.17 ± 0.09 ^c^	6.13 ± 0.68 ^B^	2.95 ± 0.14 ^a^	7.65 ± 0.41 ^A^	3.86 ± 0.07 ^d^	9.38 ± 0.46 ^D^
	Whole	3.56 ± 0.01 ^a^	8.01 ± 0.16 ^A^	3.29 ± 0.17 ^b^	15.05 ± 1.41 ^B^	2.81 ± 0.10 ^c^	4.80 ± 0.16 ^C^	3.38 ± 0.15 ^ab^	6.95 ± 0.23 ^A^	4.32 ± 0.18 ^d^	10.25 ± 1.24 ^D^
SHEEP	Skimmed	5.26 ± 0.02 ^a^	9.68 ± 0.06 ^A^	4.73 ± 0.11 ^b^	8.38 ± 0.17 ^B^	3.54 ± 0.15 ^c^	6.46 ± 0.35 ^C^	5.26 ± 0.11 ^a^	9.49 ± 0.25 ^A^	6.18 ± 0.13 ^d^	11.51 ± 0.10 ^D^
	Whole	5.28 ± 0.08 ^a^	11.09 ± 0.03 ^A^	4.73 ± 0.07 ^b^	20.25 ± 0.86 ^B^	3.83 ± 0.08 ^c^	6.46 ± 0.35 ^C^	5.22 ± 0.17 ^a^	9.44 ± 1.18 ^D^	6.71 ± 0.39 ^d^	12.08 ± 0.60 ^A^

a,b,c,d Means within a row with different lowercase superscripts indicate significant differences between milk samples.

A,B,C,D Means within a row with different uppercase superscripts indicate significant differences between retentate samples.

BCA protein determinations in milks, with or without the addition of SDS, were underestimated when compared to Kjeldahl in milk samples, with an average reduction of 11% and 33% in standard BCA and BCA+2% SDS, respectively. In whole retentate samples, the addition of SDS appeared to troubleshoot fat interference but it was not able to provide accurate results, as BCA+2% SDS underestimated protein content by 24% on average when compared to Kjeldahl values (Table 4). Skimmed retentate samples showed a similar behavior to the original milks, with an average underestimation of 15% for standard BCA and 26% for BCA+2% SDS. In contrast, the addition of 2% SDS to the Bradford assay produced a mean overestimation of 25% for milk and retentate samples.

## Conclusions

After comparative testing of the most frequently used methods to determine the total protein content, when used for milk and their retentates after UF, the Bradford assay would be the most suitable since it allows the obtention of fast and accurate results compared to the Kjeldahl reference method. Moreover, the protocol is simple to perform, requiring neither expensive equipment not experienced analysts for data acquisition. The Dumas method also provided accurate results, but it is a destructive procedure that requires a larger sample size and specific instrumentation. Regarding the BCA assay, it was significantly affected by the composition of the matrix and its use for protein quantification in dairy samples is not advised. Further research is encouraged to develop a quick routine method capable of determining trace level protein content in milk UF permeates.

## Data availability statement

The original contributions presented in the study are included in the article/supplementary material, further inquiries can be directed to the corresponding author.

## Author contributions

JF and PG-C contributed to the conception, design of the study, oversaw the research and the statistical analysis, reviewed, and edited the manuscript. DH wrote the first draft of the manuscript, carried out the laboratory work, and performed the data analysis. All authors have read and approved the final submitted version.

## Funding

This research was funded by the Spanish Ministry of Science and Innovation Projects PDC2021-121528-I00 and PDI2020-114821RB-I00 MCIN/AEI/10.13039/ 501100011033 and the Spanish National Research Council (Project 20217AT002).

## Conflict of interest

The authors declare that the research was conducted in the absence of any commercial or financial relationships that could be construed as a potential conflict of interest.

## Publisher's note

All claims expressed in this article are solely those of the authors and do not necessarily represent those of their affiliated organizations, or those of the publisher, the editors and the reviewers. Any product that may be evaluated in this article, or claim that may be made by its manufacturer, is not guaranteed or endorsed by the publisher.
